# Knockdown of lncRNA ZNRD1-AS1 suppresses gastric cancer cell proliferation and metastasis by targeting the miR-9-5p/HSP90AA1 axis

**DOI:** 10.18632/aging.203209

**Published:** 2021-07-02

**Authors:** Ming-Chen Ba, Zheng Ba, Yuan-Feng Gong, Kun-Peng Lin, Yin-Bing Wu, Yi-Nuo Tu

**Affiliations:** 1 Intracelom Hyperthermic Perfusion Therapy Center, Affiliated Cancer Hospital and Institute of Guangzhou Medical University, Guangzhou 510095, P.R. China; 2 Department of Adult Intensive Care Unit, University of Hong Kong-Shenzhen Hospital, Shenzhen 518053, P.R. China

**Keywords:** ZNRD1-AS1, gastric cancer, competitive endogenous RNA, miR-9-5p, HSP90AA1

## Abstract

LncRNAs play an important role in a variety of biological processes, such as cancer pathogenesis. The lncRNA zinc ribbon domain containing 1 antisense RNA 1 (ZNRD1-AS1) is a natural antisense transcript of ZNRD1. In this study, we found that ZNRD1-AS1 levels were significantly upregulated in gastric cancer tissues compared to those in adjacent healthy gastric tissues. ZNRD1-AS1 levels were correlated with lymph node metastasis, distal metastasis, and TNM stage, but were not correlated with age and sex. ZNRD1-AS1 knockdown suppressed cell proliferation, migration, and invasion, and promoted apoptosis. ZNRD1-AS1 overexpression had the opposite effect. ZNRD1-AS1 knockdown suppressed tumor growth and pulmonary metastasis in a nude mouse model ZNRD1-AS1 can bind to miR-9-5p and ZNRD1-AS1 knockdown can decrease the protein level of heat shock protein 90 alpha family class A member 1 (HSP90AA1), which is the target of miR-9-5p. The miR-9-5p inhibitor rescued the effect of ZNRD1-AS1 knockdown, and the mutant of miR-9-5p binding site on ZNRD1-AS1 sequence blocked the effect of ZNRD1-AS1 overexpression. In conclusion, ZNRD1-AS1 levels were upregulated in gastric cancer tissues, and knockdown of ZNRD1-AS1 suppressed gastric cancer cell proliferation and metastasis by targeting the miR-9-5p/HSP90AA1 axis. Our findings provide novel insights into the mechanism underlying the role of ZNRD1-AS1 in gastric cancer.

## INTRODUCTION

Gastric cancer is one of the most common malignant tumors of the digestive system. Its incidence is the fifth highest among malignant tumors, and the mortality rate is the third highest among cancer-related deaths in 2012 worldwide [1]. According to cancer statistics in China in 2015, the incidence (67.91/4292,000) and mortality (4.980/2.814 million) of gastric cancer both ranked second in the total incidence and death due to cancer in China [2]. Early diagnosis and effective treatment are particularly important for improving the prognosis of patients with gastric cancer. However, because the specific symptoms of gastric cancer in the early stages are not obvious, most patients develop to the advanced stage when they are first diagnosed, and the prognosis is extremely poor. Therefore, in-depth exploration of the molecular mechanisms underlying the occurrence and development of gastric cancer has important scientific significance.

Long non-coding RNAs (lncRNAs) are non-coding RNA molecules with a transcript length greater than 200 nucleotides [3]. lncRNAs can participate in the regulation of the expression of key genes at multiple levels, including epigenetic modification, transcription regulation, and post-transcriptional regulation [4]. lncRNAs play an important role in a variety of biological processes, such as cancer pathogenesis [5]. lncRNAs play a key role in regulating the growth, apoptosis, and metastasis of cancer cells [6]. The human zinc ribbon domain containing 1 (ZNRD1) antisense RNA 1 (ZNRD1-AS1) is a natural antisense transcript of ZNRD1 [7]. It plays a role by downregulating ZNRD1 [7]. lncRNAs can act as competitive endogenous RNAs (ceRNAs) to regulate the expression of key genes that participate in the regulation of tumorigenesis and development. In bladder cancer, ZNRD1-AS1 levels were upregulated, and ZNRD1-AS1 knockdown suppressed proliferation, migration, invasion, and epithelial-mesenchymal transition by functioning as a ceRNA of miR-194 [8]. In gliomas, ZNRD1-AS1 acts by sponging miR-499a-5p [9]. Recently, it was reported that ZNRD1-AS1 accelerates cell metastasis and invasion by functioning as a ceRNA of miR-335 in nasopharyngeal cancer [10]. However, there is not enough information to fully elucidate the role and mechanism of ZNRD1-AS1 in the pathogenesis of cancers. The role of ZNRD1-AS1 and its underlying mechanism in gastric cancer are unclear.

In this study, we explored the potential role of ZNRD1-AS1 in gastric cancer and elucidated its regulatory mechanism based on the ceRNA mechanism. We hypothesized that ZNRD1-AS1 might be involved in the pathogenesis of gastric cancer by functioning as a ceRNA of miR-9-5p to regulate heat shock protein 90 alpha family class A member 1 (HSP90AA1). We tested our hypothesis through *in vitro* and *in vivo* experiments. Further exploration of ZNRD1-AS1, which can be used as a ceRNA to play a biological role in tumorigenesis and development will help reveal new diagnostic and therapeutic targets.

## RESULTS

### ZNRD1-AS1 transcription is upregulated in gastric cancer tissues and cell lines

**Figure 1:**
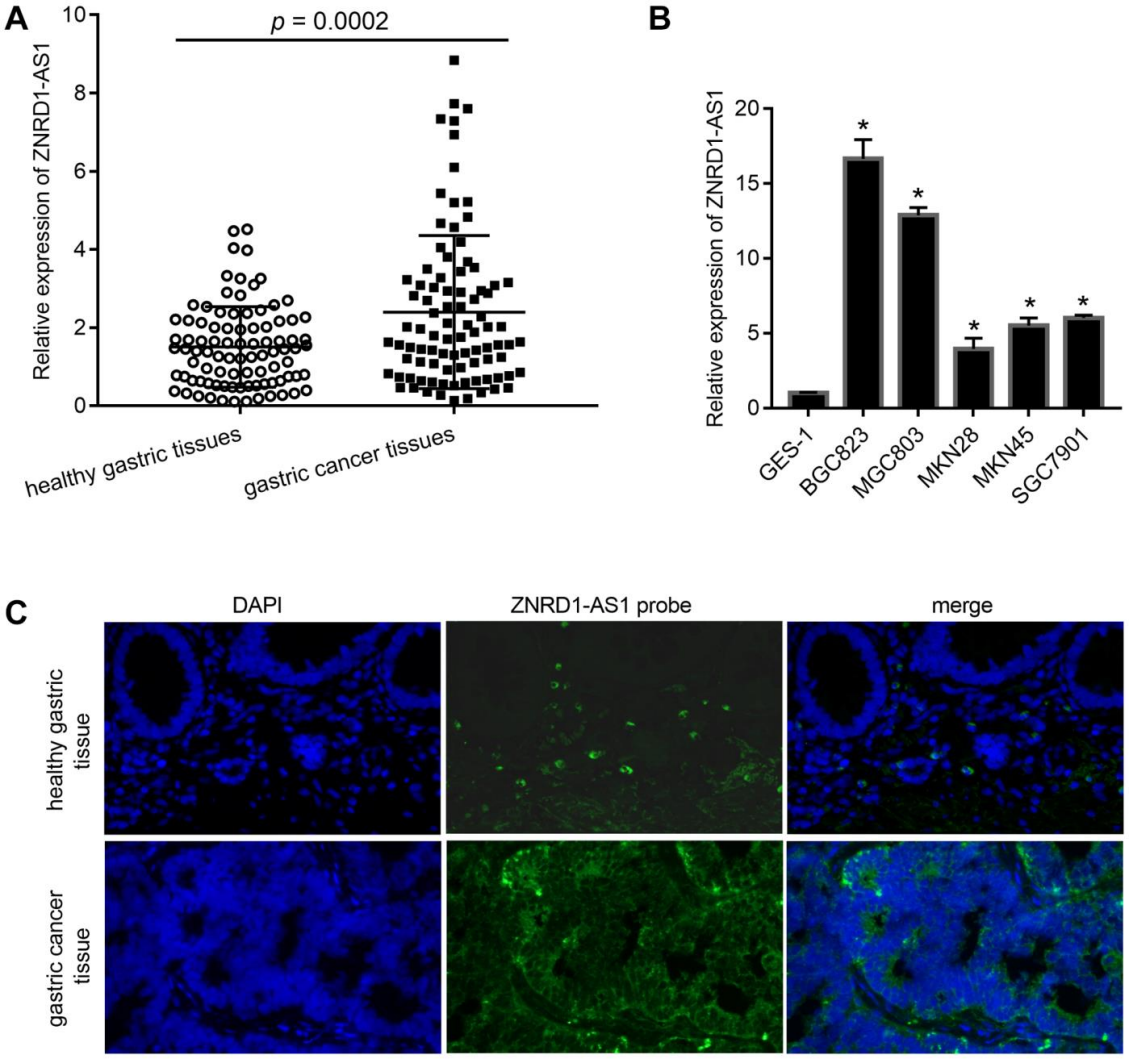
**ZNRD1-AS1 level in gastric cancer tissues and cell lines.** (**A**) ZNRD1-AS1 level in gastric cancer tissues compared to adjacent healthy gastric tissues, as detected by qRT-PCR (n=90). (**B**) ZNRD1-AS1 level in gastric cancer cell lines compared to human normal gastric epithelial cell line GES-1, as detected by qRT-PCR. *p <0.05, for gastric cancer cell line vs. GES-1. (**C**) Distribution of ZNRD1-AS1 expression in gastric cancer tissues and adjacent healthy gastric tissues (n=20).

Quantitative reverse transcription-polymerase chain reaction (qRT-PCR) showed that ZNRD1-AS1 mRNA levels were significantly increased in gastric cancer tissues relative to adjacent healthy gastric tissues (p = 0.0002) (Figure 1A). ZNRD1-AS1 mRNA levels also were upregulated in gastric cancer cell lines relative to the human normal gastric epithelial cell line GES-1 control (Figure 1B). Fluorescence *in situ* hybridization (FISH) showed that the ZNRD1-AS1 mRNA level was significantly higher in gastric cancer tissues than in adjacent healthy gastric tissues, with ZNRD1-AS1 mRNA primarily distributed in the cytoplasm (Figure 1C).

We analyzed the relationship between ZNRD1-AS1 expression and the clinicopathological characteristics of patients with gastric cancer. Patients were divided into a ZNRD1-AS1 low (less than the median) and high (more than the median) expression groups. As shown in Table 1, ZNRD1-AS1 mRNA expression levels were correlated with lymph node metastasis, distal metastasis, and TNM stage, but were not correlated with age and sex.

**Table 1:** Relationship between ZNRD1-AS1 expression and the clinicopathological characteristics of patients with gastric cancer.

**Characteristics**	**n = 90**	**ZNRD1-AS1 expression level**		***p***
		**low expression (n = 45)**	**high expression (n = 45)**	
Age				0.1864
≥ 60	58	32	26	
< 60	32	13	19	
Sex				0.5267
Male	47	22	25	
Female	43	23	20	
Tumor size				0.0568
≥ 5	49	20	29	
< 5	41	25	16	
lymphatic node metastasis				0.0118
No	35	25	15	
Yes	55	20	35	
Distal metastasis				0.0328
M0	38	24	14	
M1	52	21	31	
TNM stage				0.0459
I + II	31	20	11	
III + IV	59	25	34	

### ZNRD1-AS1 knockdown suppresses malignant characteristics and promotes apoptosis *in vitro*

**Figure 2:**
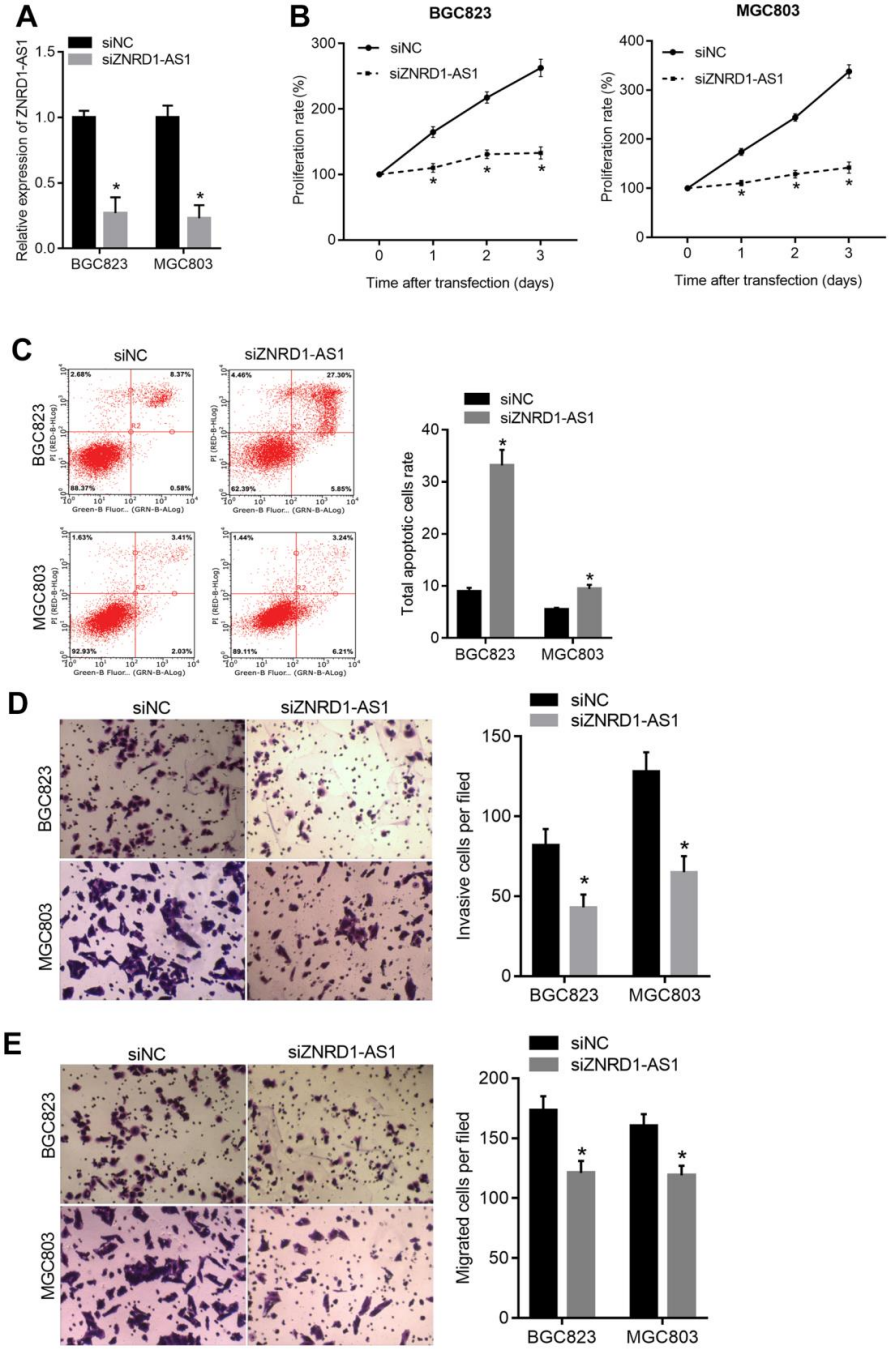
**ZNRD1-AS1 knockdown suppresses malignant characteristics and promotes apoptosis in MGC803 and BGC823 cells.** siRNA targeting ZNRD1-AS1 (siZNRD1-AS1) or negative control siRNA (siNC) was transfected into MGC803 and BGC823 cells. ZNRD1-AS1 levels were measured using qRT-PCR (**A**). Cell proliferation (**B**) and apoptosis (**C**) were analyzed by CCK8 assay and flow cytometry, respectively. Cell invasion (**D**) and migration (**E**) were analyzed by Transwell assays. Left panels show representative micrographs. Right panels show statistical results. *p <0.05 for siZNRD1-AS1 vs. siNC.

Small interfering RNA targeting ZNRD1-AS1 (siZNRD1-AS1) was transfected into MGC803 and BGC823 cells to knock down ZNRD1-AS1. As shown in Figure 2A, knockdown decreased expression relative to negative control siRNA (siNC). The proliferation of both MGC803 and BGC823 cells was decreased at 1, 2, and 3 days following siZNRD1-AS1 transfection relative to the siNC control (Figure 2B). Furthermore, the total apoptotic rate of both MGC803 and BGC823 cells was higher with siZNRD1-AS1 than with the siNC control (Figure 2C). The numbers of invasive and migrating MGC803 and BGC823 cells were lower with siZNRD1-AS1 than with the siNC control (Figure 2D, 2E). To exclude off-target effects, we assessed the effect of a second siRNA targeting ZNRD1-AS1, siZNRD1-AS1-2. As shown in Supplementary Figure 1, siZNRD1-AS1-2 had similar effects in both MGC803 and BGC823 cells. In addition, the transfection of both siZNRD1-AS1 and siZNRD1-AS1-2 increased the level of cleaved caspase 3, a marker of apoptosis (Supplementary Figure 2).

### ZNRD1-AS1 targets the miR-9-5p/HSP90AA1 axis

**Figure 3:**
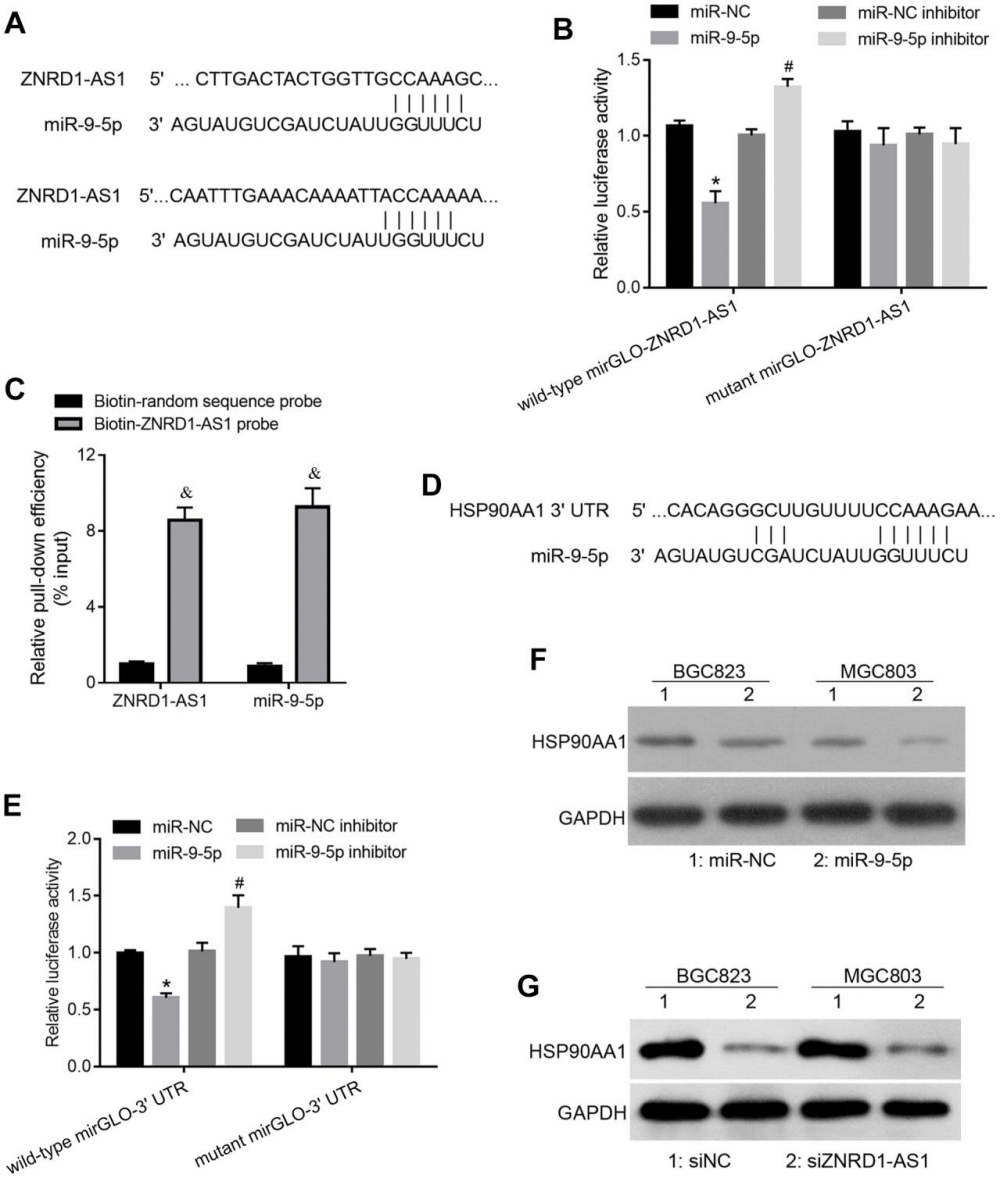
**ZNRD1-AS1 targets the miR-9-5p/HSP90AA1 axis.** (**A**) miR-9-5p binding site in the ZNRD1-AS1 sequence. (**B**) Relative luciferase activity of recombinant luciferase reporter wild-type mirGLO-ZNRD1-AS1 and mutant mirGLO-ZNRD1-AS1 in human embryonic kidney 293T cells cotransfected with miR-NC, miR-9-5p mimic, miR-NC inhibitor, or miR-9-5p inhibitor. (**C**) Relative ZNRD1-AS1 and miR-9-5p levels in RNA enriched by pulldowns. After transfection with biotinylated ZNRD1-AS1 probe (50 μM) or random sequence probe (50 μM) for 24 h, BGC823 cells were harvested for the pull-down assay. (**D**) miR-9-5p binding site on 3’ UTR of HSP90AA1. (**E**) Relative luciferase activities of recombinant luciferase reporter wild-type mirGLO-3’ UTR and mutant mirGLO-3’ UTR when cotransfected with miR-NC, miR-9-5p mimic, miR-NC inhibitor, or miR-9-5p inhibitor. (**F**) Protein expression of HSP90AA1 in MGC803 and BGC823 cells transfected with siZNRD1-AS1 or siNC, as detected by western blotting. (**G**) Protein expression of HSP90AA1 in MGC803 and BGC823 cells transfected with siZNRD1-AS1 or siNC, detected by western blotting. *p <0.05, for miR-9-5p vs. miR-NC; ^#^p <0.05, for miR-9-5p inhibitor vs. miR-NC inhibitor; ^&^p <0.05, for biotin-ZNRD1-AS1 probe vs. biotin-random sequence control probe.

To assess the regulation of ZNRD1-AS1 based on the ceRNA mechanism, we predicted the miRNA response element in the ZNRD1-AS1 sequence. Among all the predicted miRNAs, we focused on miR-9-5p, which has two potential binding sites in the ZNRD1-AS1 mRNA sequence (Figure 3A). To confirm whether miR-9-5p binds to ZNRD1-AS1, we carried out a luciferase reporter assay. As shown in Figure 3B, miR-9-5p mimic cotransfection decreased the relative luciferase activity of wild-type mirGLO-ZNRD1-AS1 compared to negative control miRNA (miR-NC) cotransfection. Cotransfection of the miR-9-5p inhibitor increased the luciferase activity of wild-type mirGLO-ZNRD1-AS1 compared to miR-NC control inhibitor cotransfection (Figure 3B). The cotransfection of miR-NC, miR-9-5p mimic, miR-NC inhibitor, or miR-9-5p inhibitor did not affect the relative luciferase activity of mutant mirGLO-ZNRD1-AS1 (Figure 3B). These results suggest that miR-9-5p can bind to the ZNRD1-AS1 sequence, which in this assay is the artificial 3’-UTR of luciferase. To confirm that ZNRD1-AS1 can function as a ceRNA of miR-9-5p in a physiological state, we carried out pulldown assays, which showed that both ZNRD1-AS1 and miR-9-5p levels were higher in RNAs enriched with the biotinylated ZNRD1-AS1 probe compared to those enriched with a biotinylated random control sequence probe (Figure 3C).

Next, we identified the targets of miR-9-5p. The binding site of miR-9-5p in the 3’-UTR of HSP90AA1 is shown in Figure 3D. Luciferase reporter assays showed that miR-9-5p mimic cotransfection decreased the relative luciferase activity of wild-type mirGLO-3’ UTR compared to miR-NC cotransfection. miR-9-5p inhibitor cotransfection increased the relative luciferase activity of wild-type mirGLO-3’ UTR compared to miR-NC inhibitor cotransfection (Figure 3E). The cotransfection of miR-NC, miR-9-5p mimic, miR-NC inhibitor, or miR-9-5p inhibitor did not affect the relative luciferase activity of the mutant mirGLO-3’ UTR (Figure 3E). These results suggest that miR-9-5p can bind to the 3’ UTR of the HSP90AA1 sequence, which is the artificial 3’ UTR of luciferase. Moreover, we found that miR-9-5p overexpression decreased HSP90AA1 protein levels compared to miR-NC in HSP90AA1 cells (Figure 3F).

We found that the HSP90AA1 protein expression was lowered by siZNRD1-AS1 group relative to the siNC control (Figure 3G), suggesting that ZNRD1-AS1 can regulate HSP90AA1.

### miR-9-5p inhibition rescues ZNRD1-AS1 knockdown in cultured cells

**Figure 4:**
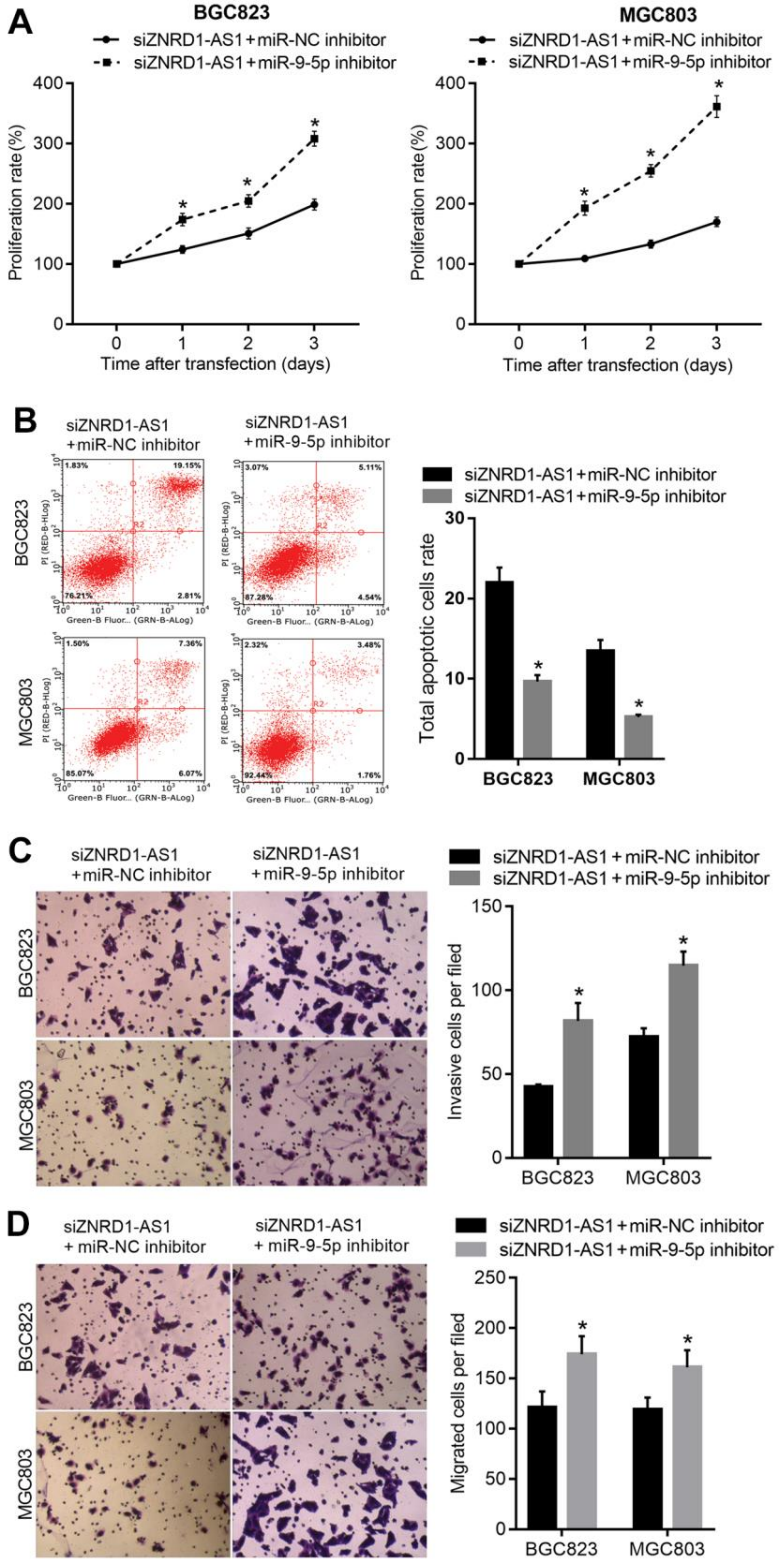
**miR-9-5p inhibitor rescues the effect of ZNRD1-AS1 knockdown on malignant characteristics and apoptosis.** miR-9-5p inhibitor was cotransfected into MGC803 and BGC823 cells; with cotransfection of siZNRD1-AS1 and miR-NC inhibitor as control. Cell proliferation (**A**) and apoptosis (**B**) were analyzed by CCK8 assays and flow cytometry, respectively. Cell invasion (**C**) and migration (**D**) were analyzed by Transwell assays. Left panels show representative micrographs. Right panels show statistical results. *p <0.05, for siZNRD1-AS1 + miR-9-5p inhibitor vs. siZNRD1-AS1 + miR-NC inhibitor.

To determine whether ZNRD1-AS1 functions as a ceRNA of miR-9-5p, the miR-9-5p inhibitor was cotransfected into MGC803 and BGC823 cells with siZNRD1-AS1 to rescue the effect of ZNRD1-AS1 knockdown on miR-9-5p. Cotransfection of siZNRD1-AS1 and miR-NC inhibitor was used as a control. The proliferation was increased (Figure 4A), the total apoptotic rate was decreased (Figure 4B), and the numbers of invasive and migratory cells were increased (Figure 4C, 4D).

### Mutating miR-9-5p binding site in ZNRD1-AS1 mRNA partially blocks the effects of ZNRD1-AS1 overexpression

**Figure 5:**
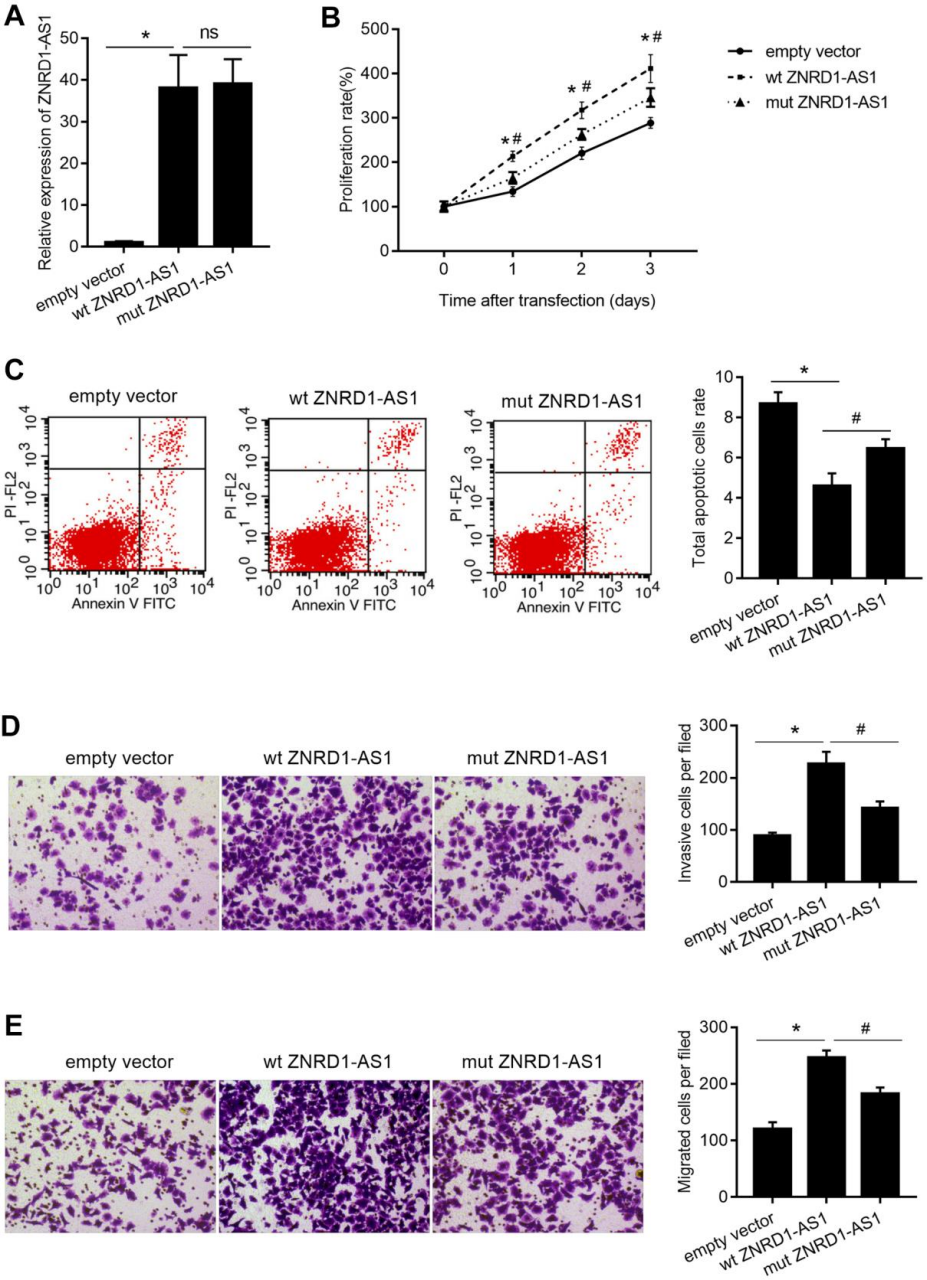
**Effect of wt ZNRD1-AS1 or mut ZNRD1-AS1 overexpression on MKN28 cells.** Wild-type and mutant ZNRD1-AS1 constructs or an empty vector were transfected into MKN28 cells. ZNRD1-AS1 mRNA expression was measured by qRT-PCR (**A**). Cell proliferation (**B**) and apoptosis (**C**) were analyzed using the CCK8 assay and flow cytometry, respectively. Cell invasion (**D**) and migration (**E**) were analyzed using the transwell assay. Left panels show representative micrographs. Right panels show statistical results. *p <0.05, for empty vector vs. wt ZNRD1-AS1; ^#^p <0.05, for mut ZNRD1-AS1 vs. wt ZNRD1-AS1; ns, p >0.05.

**Figure 6:**
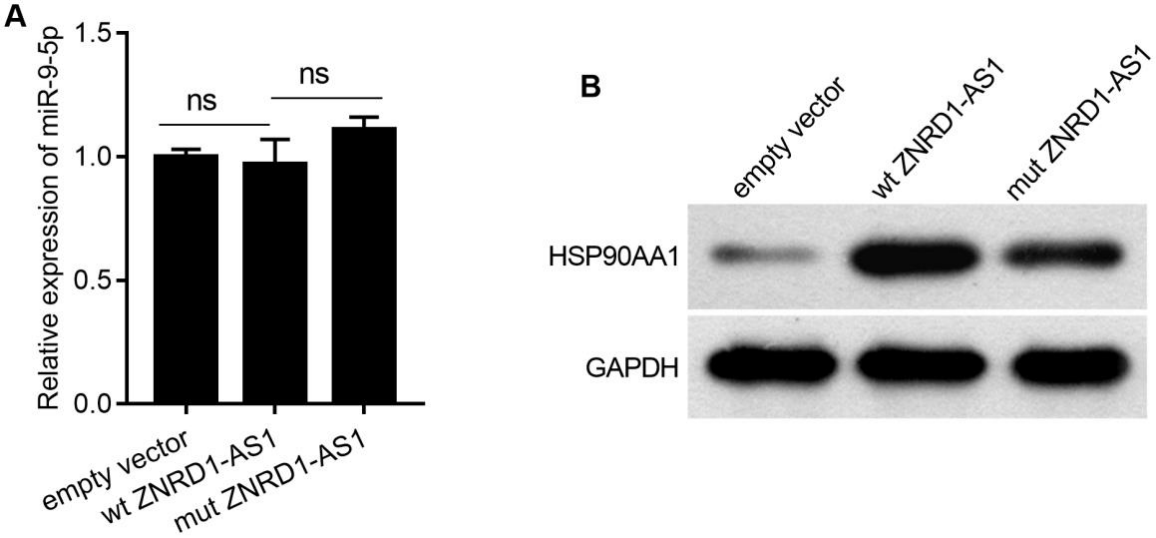
**Effect of wt ZNRD1-AS1 or mut ZNRD1-AS1 overexpression on miR-9-5p or HSP90AA1 protein levels in MKN28 cells.** Wild-type ZNRD1-AS1 or ZNRD1-AS1 with mutated miR-9-5p binding sites, with an empty-vector control were transfected into MKN28 cells. miR-9-5p levels was measured using qRT-PCR (**A**). HSP90AA1 protein expression analyzed by western blot (**B**). ns, p >0.05.

As shown in Figure 5A, wild-type (wt) ZNRD1-AS1 or ZNRD1-AS1 with mutated miR-9-5p binding sites (mut ZNRD1-AS1) was overexpressed in MKN28 cells. In contrast to the effect of ZNRD1-AS1 knockdown, both wt and mut ZNRD1-AS1 overexpression promoted cell proliferation, migration, and invasion, as well as inhibited apoptosis in MKN28 cells (Figure 5). Compared to wt ZNRD1-AS1, proliferation was lower (Figure 5B), the total apoptotic rate was higher (Figure 5C), and the numbers of invasive and migratory cells were lower (Figure 5D, 5E) with mut ZNRD1-AS1. In addition, neither wt nor mut ZNRD1-AS1 overexpression affected the levels of miR-9-5p in MKN28 cells (Figure 6A). Both wt and mut ZNRD1-AS1 overexpression increased HSP90AA1 protein levels, which were decreased by mut ZNRD1-AS1 relative to wt ZNRD1-AS1 (Figure 6B).

### ZNRD1-AS1 knockdown suppresses tumor growth and pulmonary metastasis in a nude mouse model

**Figure 7:**
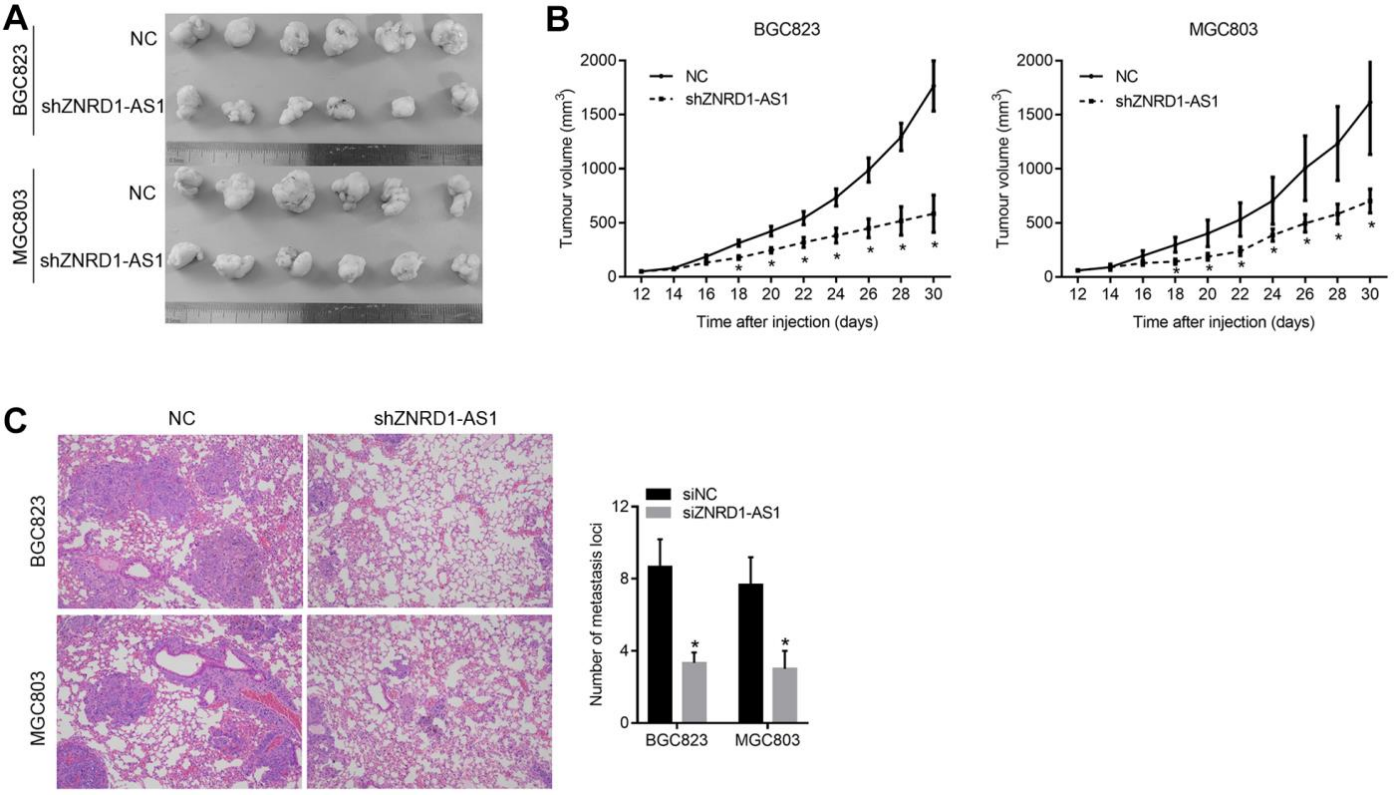
**ZNRD1-AS1 knockdown suppresses tumor growth and pulmonary metastasis in a nude mouse model.** (**A**) Micrographs of subcutaneous tumors formed by MGC803 and BGC823 cells of the shZNRD1-AS1 and NC groups. (**B**) Growth curves of subcutaneous tumors formed by MGC803 and BGC823 cells of the shZNRD1-AS1 or NC groups. (**C**) Metastasis in the lungs of nude mice intravenously injected with MGC803 and BGC823 cells from the shZNRD1-AS1 or NC groups. Left panels show representative micrographs. The right panel is a graph of mean metastatic loci.

To confirm the effect of ZNRD1-AS1 knockdown *in vivo*, we constructed a subcutaneous xenograft and pulmonary metastasis model in nude mice. We found that the volumes of subcutaneous tumors formed by MGC803 and BGC823 cells treated with shZNRD1-AS1 were smaller than those with NC (Figure 7A, 7B). Moreover, we found that there were more and larger metastases in the lung tissues of nude mice intravenously injected with MGC803 and BGC823 cells with shZNRD1-AS1 than with NC (Figure 7C).

## DISCUSSION

In cancer pathogenesis, lncRNAs have been shown to exhibit tumor-suppressive and tumor-promoting functions [6]. In the present study, we investigated the function and regulation of ZNRD1-AS1 in gastric cancer. We found that ZNRD1-AS1 levels were significantly upregulated in gastric cancer, both in tissues and cell lines. This result is consistent with those reported previously. ZNRD1-AS1 also has higher expression levels in lung, bladder, and nasopharyngeal cancers [8, 10]. These data suggest that ZNRD1-AS1 promotes the progression of gastric cancer. The correlation between ZNRD1-AS1 levels and clinicopathological characteristics, including lymph node metastasis, distal metastasis, and TNM stage, further supports this hypothesis. Moreover, this hypothesis was tested by functional experiments both in cell culture and *in vivo*.

We found that ZNRD1-AS1 knockdown suppressed cell proliferation, migration, and invasion, and promoted apoptosis in MGC803 and BGC823 cells. In addition, ZNRD1-AS1 knockdown suppressed subcutaneous tumor growth and pulmonary metastasis in a nude mouse model. Moreover, ZNRD1-AS1 overexpression promoted cell proliferation, migration, and invasion, and inhibited apoptosis in the gastric cancer cell line MKN28. These results are consistent with a hypothesized role for ZNRD1-AS1 in gastric cancer. Our conclusion is supported by similar functions of ZNRD1-AS1 in other types of cancer [7–11]. Together, these data identify ZNRD1-AS1 as a potential therapeutic target.

Both luciferase reporter and pulldown assays demonstrated that ZNRD1-AS1 can bind to miR-9-5p. In addition, the rescue by miR-9-5p inhibitor of the effect of ZNRD1-AS1 knockdown and the inhibition by the miR-9-5p binding-site mutant of the effect of ZNRD1-AS1 overexpression further demonstrated that ZNRD1-AS1 may act as a buffer for miR-9-5p. This hypothesis is further supported by the suppressive effect of miR-9-5p on the proliferation, migration, and invasion of gastric cancer cells [12, 13]. However, the ZNRD1-AS1 sequence has many miRNA response elements, suggesting that ZNRD1-AS1 may play a role as a ceRNA of other miRNAs. It has been identified as a ceRNA of miR-194, miR-335, and miR-499a-5p [8–10]. In addition, ZNRD1-AS1 is an antisense transcript of ZNRD1, and it plays a role in ZNRD1 expression [14]. Therefore, it appears to be impossible for ZNRD1-AS1 to exert its regulatory role only by sponging miR-9-5p. The partial block of the miR-9-5p binding site mutant on the effect of ZNRD1-AS1 overexpression can further support this hypothesis. In the present study, we did not explore the comprehensive regulatory network underlying ZNRD1-AS1, but we identified a new mechanism underlying its function in gastric cancer.

The regulatory network of lncRNAs that act as ceRNAs includes lncRNAs, miRNAs, and the target mRNAs of miRNAs [15, 16]. Therefore, it is necessary to identify the target mRNA of miR-9-5p to elucidate the regulatory mechanism of ZNRD1-AS1. In this study, we focused on HSP90AA1, also known as HSP90alpha, an isoform of the molecular chaperone HSP90 [17]. It is a member of the heat shock gene family, which is constitutively expressed in cells and inducible under conditions such as heat stress [18]. Functional studies in multiple types of cancers have shown that HSP90 can promote cancer progression and induce drug resistance; therefore, its inhibition may be a promising strategy for cancer therapy [19]. Moreover, high expression of HSP90AA1 is associated with disease progression and poor survival in patients with gastric cancer [20–22]. We showed that miR-9-5p binds to the 3’ UTR of HSP90AA1 mRNA, and miR-9-5p decreased HSP90AA1 protein expression. Moreover, we found that ZNRD1-AS1 knockdown decreased HSP90AA1 protein expression. These results suggest that ZNRD1-AS1 regulates HSP90AA1 expression by functioning as a ceRNA of miR-9-5p. Based on the role of HSP90AA1 in cancer progression, we hypothesized that ZNRD1-AS1 may play a role through the miR-9-5p/HSP90AA1 axis. However, we found that the effect of ZNRD1-AS1 silencing on HSP90AA1 protein expression (Figure 3G) was much more pronounced than the overexpression of miR-9 mimics (Figure 3F). These data suggest that additional mechanisms are involved in the regulation of HSP90AA1 by ZNRD1-AS1. In addition, miR-9-5p also has a binding site on the 3’-UTR of other mRNAs; Hence, the ZNRD1-AS1/miR-9-5p axis may play a role in targeting other genes.

In conclusion, ZNRD1-AS1 levels were upregulated in gastric cancer tissues, with ZNRD1-AS1 knockdown suppressed gastric cancer cell growth and metastasis in cultured cells and *in vivo*. ZNRD1-AS1 may play an important role in the regulation of the miR-9-5p/HSP90AA1 axis. However, this mechanism cannot expound the comprehensive regulatory network underlying ZNRD1-AS1; therefore, additional mechanisms may be involved in its regulation in gastric cancer progression. Our findings identify lncRNA ZNRD1-AS1 as a potential therapeutic target for gastric cancer.

## MATERIALS AND METHODS

### Cancer tissue specimens

Patients with gastric cancer (n = 90) were enrolled between January 2017 and December 2018 at the Affiliated Tumor Hospital of Guangzhou Medical University. Tumor tissues and healthy gastric tissues adjacent to them were collected after surgery. All patients understood the purpose of the experiment and the usefulness of the specimens. Written consent was obtained from all patients. General, clinical, and pathological data were recorded in detail. This study was approved by the Ethics Committee for Medical Research of the Affiliated Tumor Hospital of Guangzhou Medical University, in accordance with the Declaration of Helsinki.

### FISH

Twenty pairs of tumor tissues and healthy gastric tissues adjacent to them were randomly selected from specimens for ZNRD1-AS1 detection using qRT-PCR. FISH was performed on formalin-fixed, paraffin-embedded sections as described by Lim et al. [23]. The probe for ZNRD1-AS1 was 5’-FAM-AGACTCTGCTTTGGCAAGCCA-FAM-3’.

### Cell culture

The normal human gastric epithelial cell line GES-1 and human gastric cancer cell lines (MKN28, MKN45, and SGC7901) were purchased from the Institute of Biochemistry and Cell Biology, SIBS, CAS (Shanghai, China). Human gastric cancer cell lines MGC803 and BGC823 were purchased from the American Type Culture Collection (Manassas, VA, USA). MGC803 cells were cultured in Dulbecco’s modified Eagle’s medium with 10% fetal bovine serum; GES-1, MKN28, MKN45, SGC7901, and BGC823 were cultured in RPMI 1640 medium with 10% fetal bovine serum at 37° C in a 5% CO_2_ incubator.

### siRNA, miRNA mimic, ZNRD1-AS1 overexpression plasmid

The siRNAs siZNRD1-AS1 and siZNRD1-AS1-2 were designed by Shanghai GenePharma Co., Ltd. (China). The sense sequences were GCGUUAGAGAUGGAGACUATT-3’ and 5’-GCAACCGGCCUCGUGCUUUTT-3’, respectively. The sense sequence of siNC was 5’-UUCUCCGAACGUGUCACGUTT-3’. The miR-9-5p mimic, miR-9-5p inhibitor, miR-NC, and miR-NC inhibitor were synthesized by Shanghai GenePharma. The sequence of wild-type ZNRD1-AS1 (NR_145418) was cloned into the pcDNA3.1 plasmid (Promega, Madison, WI, USA) using DNA synthesis *in vitro* by GENEWIZ Co., Ltd, resulting in the construct wt ZNRD1-AS1. The binding sites of miR-9-5p in wt ZNRD1-AS1 (Figure 4A) were mutated using the Site-Directed Mutagenesis Kit (Stratagene, San Diego, CA, USA); the resulting construct was designated as mut ZNRD1-AS1. The empty pcDNA3.1 vector was abbreviated as an empty vector. The CCAAAG sequence of the miR-9 binding sites were mutated to GGTAAC.

### Cell transfection

siNC, siZNRD1-AS1, siZNRD1-AS1-2, siZNRD1-AS1 + miR-NC inhibitor, siZNRD1-AS1 + miR-9-5p inhibitor, empty vector, wt ZNRD1-AS1, or mut ZNRD1-AS1, were transfected into cells at 70% confluence using Lipofectamine™ RNAiMAX (Invitrogen).

### qRT-PCR

Total RNA from clinical tissues and cultured cells was isolated using TRIzol (Invitrogen, Carlsbad, CA, USA). Reverse transcription (RT) was performed to obtain cDNA using M-MLV reverse transcriptase (Promega). The RT primer for the ZNRD1-AS1 analysis was Oligo d(T). The RT primer for miR-9-5p analysis was a stem-loop primer (sequence: 5’-CTCAACTGGTGTCGTGGAGTCGGCAATTCAGTTGAGTCATACAG-3’). PCR was carried out using SYBR Green qPCR SuperMix (Invitrogen). PCR results were analyzed using the 2^−ΔΔCt^ method to quantify relative RNA expression levels. The forward and reverse PCR primers for ZNRD1-AS1 were 5’-CGTACGTTGCCAAATAATGA and 5’-ATACGTCCCAGAACACTTAG, respectively. The forward and reverse PCR primers for miR-9-5p were 5’-ACACTCCAGCTGGGTCTTTGGTTATCTAGCTGTATG-3’ and 5’-CTCAACTGGTGTCGTGGA-3’, respectively.

### Cell proliferation assays

The Cell Counting Kit-8 (CCK8, Beyotime Institute of Biotechnology, Shanghai, China) was used to measure cell proliferation. MGC803 and BGC823 cells (1 × 10^4^ per well) were cultured in 96-well plates. CCK8 solution (10 μL) was added to each well after transfection and incubation for 24, 48, and 72 h. After incubation for 4 h, the optical density (OD) at 450 nm was measured using a Multiskan MK3 microplate reader (Thermo Fisher Scientific, Waltham, MA, USA). The proliferation rate was calculated based on OD_450 nm._ using the formula: proliferation rate = OD_450 nm_ value of other time point ÷ OD_450 nm_ value of 0 d ×100% (same group).

### Apoptosis assay

After transfection for 48 h, cells were harvested for flow cytometry and treated using the Annexin V-FITC/PI Apoptosis Detection Kit (Jiangsu Keygen Biotechnology Co. Ltd., Nanjing, China), according to the manufacturer’s instructions. Apoptosis levels were measured by flow cytometry. The total apoptotic cell rate is the sum of the upper right quadrant and lower right quadrant of the flow cytometric quadrantal diagram.

### Migration assays

After transfection for 24 h, cells were harvested for Transwell assay to measure migration and invasiveness. After counting, 1 × 10^5^ cells in 200 μL serum-free culture medium were added to the upper chamber. Culture medium containing 10% fetal bovine serum (600 μL) was added to the lower chamber. For the invasion assay, the upper chamber was pre-coated with Matrigel (BD Biosciences). After incubation at 37° C in a 5% CO_2_ incubator for 24 h, nonmigrating or invading cells in the upper chamber were wiped with a cotton swab. After washing twice with PBS, these cells were fixed with 4% paraformaldehyde for 15 min. After washing twice with PBS, cells were stained with 0.5% crystal violet for 10 min and then washed twice with PBS. Finally, pictures of five random fields were taken with a microscope (Olympus, Tokyo, Japan). Average counts of cells were used. Each experiment was conducted in triplicate.

### Western blotting

Total protein isolation, protein concentration quantification, and western blotting were performed according to a previously reported method [24]. The catalog numbers and dilutions of primary antibodies were: HSP90AA1 (ab240899, 1:1000), cleaved caspase-3 (ab2302, 1:500), and GAPDH (ab9485, 1:2500). GAPDH was used as a loading control. All antibodies were purchased from Abcam (Cambridge, MA, USA).

### Luciferase reporter assays

To create a reporter, the full-length ZNRD1-AS1 sequence or the 3’ UTR of HSP90AA1 was cloned into the dual-luciferase miRNA target expression vector GP-mirGLO (Promega) as artificial 3’ UTRs. The resulting plasmids were designated as wild-type mirGLO-ZNRD1-AS1 and wild-type mirGLO-3’ UTR, respectively. The binding sites of miR-9-5p in mirGLO-ZNRD1-AS1 (two sites shown in Figure 4A) or wild-type mirGLO-3’ UTR (one site shown in Figure 4D) were mutated using the Site-Directed Mutagenesis Kit (Stratagene), producing constructs designated as mutant mirGLO-ZNRD1-AS1 and mutant mirGLO-3’ UTR, respectively. The CCAAAG sequence of the miR-9 binding site was mutated to GGTAAC. All reporter plasmids were sequenced.

Human embryonic kidney 293T cells were seeded in 96-well plates. When the confluence reached 60%, cells were cotransfected with one of the above luciferase reporters and the miR-NC or miR-9-5p mimic. After incubation for 48 h, Ranilla luciferase and firefly luciferase were measured using Dual-Glo Luciferase Assay reagents (Promega). All assays were conducted in triplicate.

### Pulldown assay

BGC823 cells were seeded in a 6-cm Petri dish. When the confluence reached 60% (approximately 2 × 10^6^ cells), cells were transfected with a biotinylated ZNRD1-AS1 probe (50 μM) or a control random sequence probe (50 μM). At 24 h after transfection, cells were harvested and pulldowns were carried out using the method described by Phatak and Donahue [25]. Levels of ZNRD1-AS1 and miR-9-5p in the enriched RNAs were determined using qRT-PCR.

### Subcutaneous xenograft and pulmonary metastasis model in nude mice

All animal experiments were approved by the Institutional Animal Care and Use Committee of Guangzhou Medical University (No.GD2019-040). A short hairpin RNA (shRNA) that could be cut into siRNAs targeting ZNRD1-AS1, named shZNRD1-AS1, was designed using the sequence of siZNRD1-AS1. Lentiviruses expressing shZNRD1-AS1 or empty controls were purchased from Genechem (Shanghai, China). ZNRD1-AS1 levels in MGC803 and BGC823 cells were stably knocked down by infecting cells with lentiviruses expressing shZNRD1-AS1. Cells infected with the empty lentivirus were used as negative controls (NC).

To construct a subcutaneous xenograft model, twenty-four six-week-old athymic nude mice were divided into four groups (n = 6): MGC803 shZNRD1-AS1, MGC803 NC, BGC823 shZNRD1-AS1, and BGC823 NC. Cells (3 × 10^6^) in 0.2 mL PBS were subcutaneously injected into the right armpit region. At 12, 14, 16, 18, 20, 22, 24, 26, 28, and 30 days following injection, the tumor length (L, the maximum transcutaneous diameter), and width (W, the corresponding maximum vertical transcutaneous diameter) of each tumor were measured using Vernier calipers. The tumor growth curve was based on the value of the tumor volume calculated using the formula: ½ × L × W^2^ [26].

To construct a pulmonary metastasis model, six-week-old athymic nude mice were divided into four groups as described below (n = 4). Cells (5 × 10^5^) were resuspended in 100 μL of Hank’s balanced salt solution and injected intravenously through the tail. Six weeks later, all mice were euthanized by intraperitoneal injection of sodium pentobarbital (130 mg/kg). Lung tissues were isolated for hematoxylin and eosin (H&E) staining to analyze the formation of metastatic foci.

### Hematoxylin-eosin (H&E) staining

Staining was performed according to the instructions of the kit purchased from Abcam (Cambridge, MA, USA). Six micrographs were randomly taken from each section, then the number of metastatic foci were counted. Mean counts of metastatic foci in each are presented.

### Statistical analyses

All statistical analyses were performed using GraphPad Prism version 7.0 (GraphPad Software, San Diego, CA, USA). The Mann-Whitney test was used for statistical analysis of ZNRD1-AS1 expression in gastric cancer tissues and adjacent healthy tissues. The significance of the relationship between ZNRD1-AS1 expression and the clinicopathological features of patients with gastric cancer was determined using the chi-square test. An unpaired *t*-test was used for statistical analysis between the two groups. Statistical significance was set at *P* <0.05.

### Availability of data and materials

All data from this study are available in this published article.

### Ethics approval and consent to participate

Written informed consent was obtained from all participants. Our study was approved by the Ethics Committee for Medical Research of the Affiliated Tumor Hospital of Guangzhou Medical University, in accordance with the Declaration of Helsinki.

## Supplementary Material

Supplementary Figures
