# Supplementary material for: Knockdown of lncRNA ZNRD1-AS1 suppresses gastric cancer cell proliferation and metastasis by targeting the miR-9-5p/HSP90AA1 axis
Source: Aging (Albany NY). 2021 Jul 2;13(13):17285–301. doi: 10.18632/aging.203209 (PMC8312431; doi:10.18632/aging.203209)
Supplement: Supplementary Figures [file aging-13-203209-s001.pdf]

SUPPLEMENTARY FIGURES

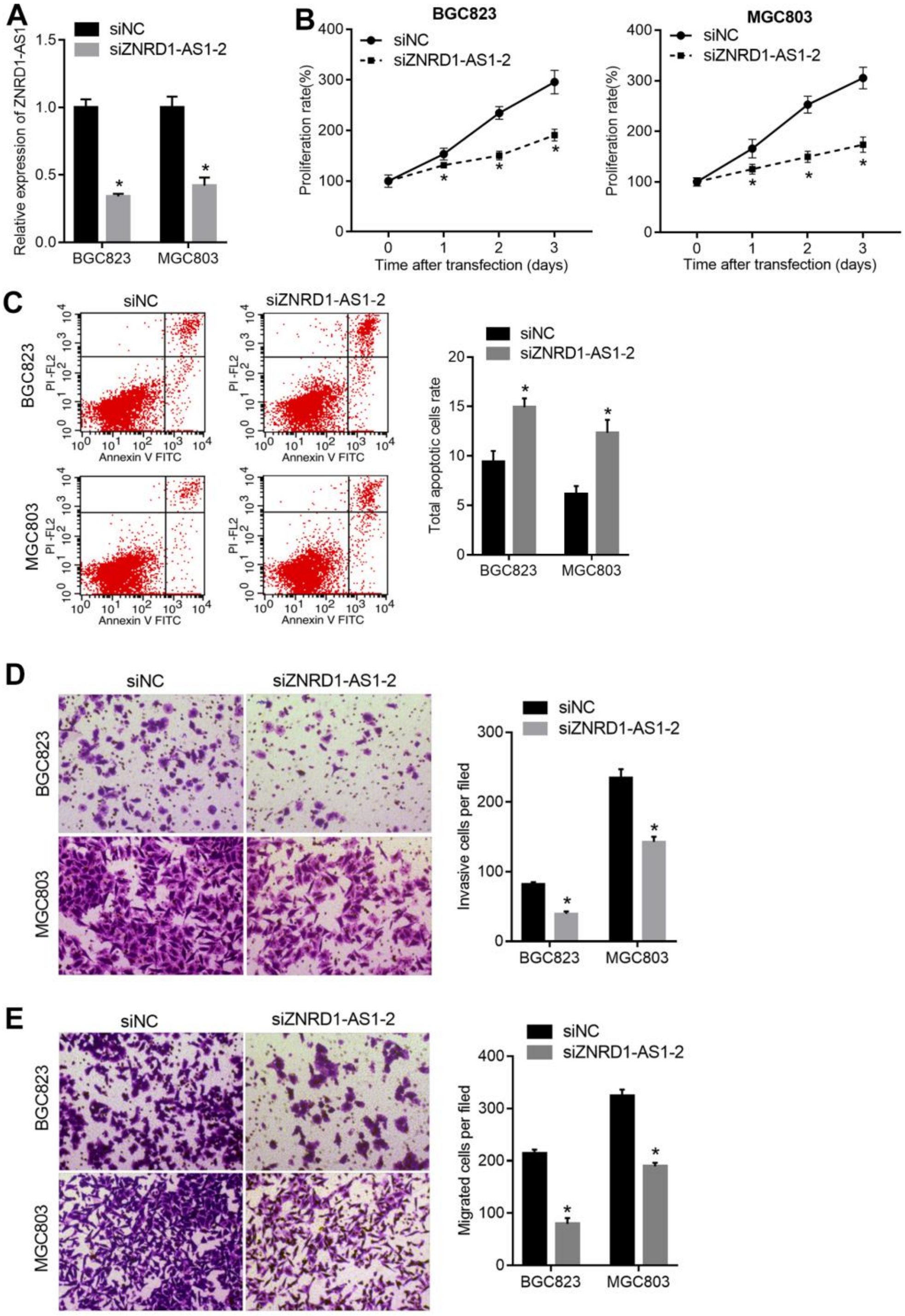

Supplementary Figure 1. Transfection of siZNRD1-AS1-2 suppresses malignant properties and promotes apoptosis in MGC803 and BGC823 cells. siRNA-2 targeting ZNRD1-AS1 (siZNRD1-AS1-2) or negative control siRNA (siNC) was transfected into cells. ZNRD1-AS1 mRNA expression levels were measured using qRT-PCR (**A**). Cell proliferation (**B**) and apoptosis (**C**) were analyzed by CCK8 assay and flow cytometry, respectively. Cell invasion (**D**) and migration (**E**) were analyzed by Transwell assays. Left panels show representative micrographs. Right panels show statistical results. *p <0.05, for siZNRD1-AS1-2 vs. siNC.

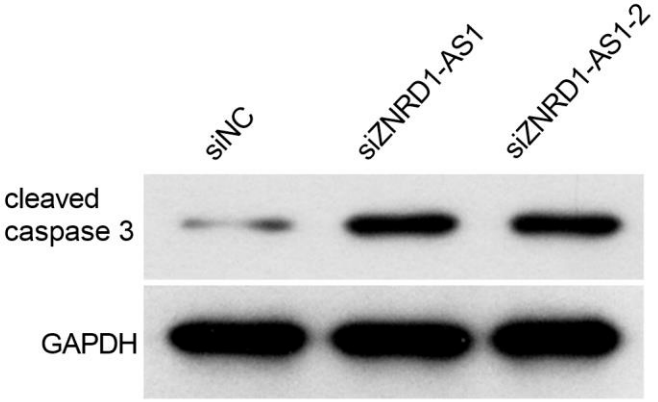

Supplementary Figure 2. Effect of ZNRD1-AS1 knockdown on cleaved caspase 3 levels in MGC803 cells. Two siRNAs targeting ZNRD1-AS1 (named as siZNRD1-AS1 or siZNRD1-AS1-2) or negative control siRNA (siNC) were transfected into MGC803; cleaved caspase 3 levels were measured by western blot.
